# Early Life Management of Osteogenesis Imperfecta

**DOI:** 10.1007/s11914-023-00823-5

**Published:** 2023-09-26

**Authors:** Paul Arundel, Stephanie A. Borg

**Affiliations:** 1 Sheffield Children’s NHS Foundation Trust, Sheffield, UK; 2 Highly Specialised Service for Complex Childhood Osteogenesis Imperfecta, NHS England, London, UK

**Keywords:** Osteogenesis imperfecta, Infant, Bisphosphonate, Rehabilitation, Management, Type 1 collagen

## Abstract

**Purpose of Review:**

This review aims to provide a review of the multidisciplinary management of infants with osteogenesis imperfecta (OI) during the first year of life, focusing on those with severe disease. The authors draw on published literature and direct experience of working in a large paediatric centre specialising in the management of rare bone disease.

**Recent Findings:**

Whilst understanding of the pathophysiology of OI has grown over the past decade, the evidence base for management of infants remains limited. There has been a greater recognition of certain subjects of concern including pain management, cervical spine deformity, and neurocognitive development. Both international consensus guidelines on rehabilitation and disease-specific growth charts have been welcomed by clinical teams.

**Summary:**

The early involvement of multidisciplinary specialist care is critical in ensuring optimal care for the infant with severe OI. A long-term perspective which focuses on the axial, craniofacial, and peripheral skeleton as well as on development more generally provides a framework which can guide the management of infants with severe OI.

## Introduction

Osteogenesis imperfecta (OI) is a disease characterised by bone fragility, with a spectrum of disease in infancy ranging from those with so called “mild”, type 1 disease in whom there may be no obvious or unequivocal clinical features of disease to severe, type 3 disease with obvious manifestations including multiple fractures and long bone deformities. Most cases are caused by dominant mutations in the type 1 collagen genes, *COL1A1* and *COL1A2*. However, in severe cases, there is a higher incidence of homozygous recessive mutations in a variety of other genes involved in type 1 collagen production, processing, and trafficking [1]. As understanding of genotype–phenotype relationships grows and treatments evolve, it is likely that the genotype of an individual case will become increasingly important in determining care. At present, it is generally that case that management of an infant with OI is determined by the severity of the clinical phenotype, rather than the underlying genotype. This article focuses mainly on the management of infants with severe OI.

## Typical Presentations of An Infant with Osteogenesis Imperfecta

Cases of severe OI are commonly identified antenatally with imaging showing reduced growth, and short, deformed, or fractured limbs [1]. In such situations, there should already have been discussions between the fetomaternal, neonatal, and paediatric bone services resulting in a clear and written plan for delivery and immediate care in the neonatal period. Discussion should also have involved the family, with care taken to ensure that they have understood what is likely to happen. It is important that delivery takes place in a centre with the neonatal facilities and expertise capable of dealing with the complexity and range of problems that may arise. These include respiratory insufficiency, which is the main cause of death in the first few months of life. It is helpful if, prior to delivery, the family have met the clinicians who will be responsible for the care of the infant after birth, in order that some mutual understanding and trust may be in place in advance of the challenges of the neonatal situation.

Cases of less severe OI are often identified through a family history. In these situations, an infant will commonly have no obvious or unequivocal features of disease. Here, the task of the clinician is to explain any uncertainty to the family and to try and resolve it. If a familial pathogenic mutation is known, then screening of the infant for the same mutation may take place. Alternatively, should this not be an option, or the family not wish to undertake genetic screening, then a plan for clinic follow-up should be made with an open invitation to contact should any concerns arise. This can be particularly important in the situation of subsequent unexplained fractures; timely involvement of a clinician who knows the family can expedite investigations and potentially avoid unnecessary separation of children and families should abuse be wrongly suspected.

Of course, a child may be diagnosed with OI through investigation of unexplained fractures in the absence of a family history. Whilst the differentiation of OI from abusive injury is beyond the scope of this article, it is worth noting that the impacts on families of the processes around the investigation of unexplained fractures can be profound and should be anticipated by the specialist team with extra support and early offers of contact with a psychologist.

Even in the absence of a clear diagnosis, it is prudent to advise careful handling of infants suspected of having OI. Advice to clinicians includes the avoidance of unmodified routine checks by inexperienced staff. We generally advise assessment of the hips by an experienced clinician (typically an orthopaedic surgeon), usually alongside ultrasound of the hips. Advice to parents will include the use of relatively loose clothing and avoidance of forceful, particularly twisting, movements during handling, such as when changing nappies, or manipulating limbs into clothing [2].

## Early Postnatal Management of the Infant with Severe Osteogenesis Imperfecta

The immediate postnatal management of an infant with severe osteogenesis imperfecta can be difficult and is a period of stress and anxiety for both parents and neonatal teams inexperienced in the care of such infants. These difficulties can be mitigated considerably by a clear plan having been made in the antenatal period and the early involvement of a specialist team dedicated to the care of children with rare bone disease. These steps depend on clear pathways of care with well-established and open communication between neonatal teams and their local specialist services.

**Fig. 1:**
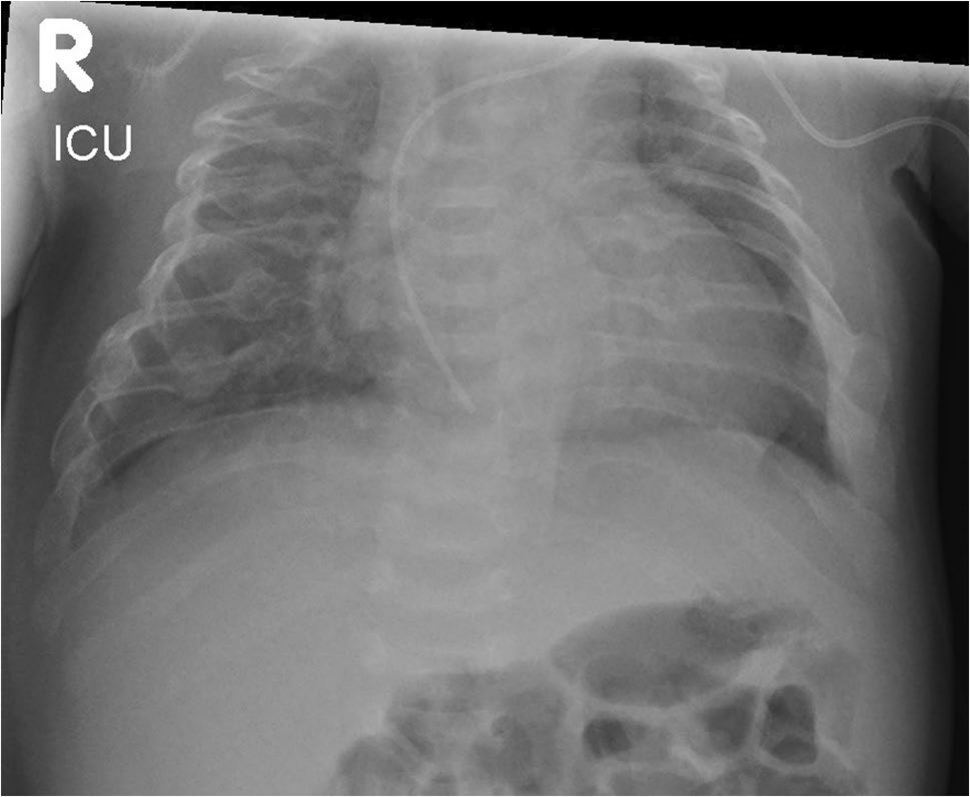
Plain radiograph of chest of an infant with severe osteogenesis imperfecta on a neonatal intensive care unit. There are multiple rib and vertebral fractures with some reduction in chest size. The infant was self-ventilating in low-flow oxygen at the time of the radiograph and did briefly show signs of respiratory failure on blood gas measurements but did not require continuous positive pressure support

Following delivery, assessment should focus on basic life support, i.e. establishing airway, breathing, and circulation. In severe cases of OI, a small chest, pulmonary hypoplasia, intrinsic lung disease, airway collapse, and multiple fractures, potentially with flail segments, can all significantly impair an infant’s ability to effectively self-ventilate (Fig. 1). Pain from fractures is another important element.

Severe osteogenesis imperfecta itself is part of a spectrum of disease extending from lethal disease which is incompatible with life to infants with long bone deformities but no difficulties of self-ventilation. It is sometimes difficult to predict the precise severity and cause of disease based on antenatal evidence [3]. Thus, it is prudent to obtain a postnatal assessment by a clinical team experienced in OI at the earliest opportunity to help guide care. Indicators of severity relevant to the assessment of lethality include a small chest/pulmonary hypoplasia, “beading” of ribs, and marked shortening and angulation of long bones. Sadly, there are situations in which disease is so severe that the infant is unlikely to survive, even with the best care. In these situations, the usual ethical and legal principles apply. Clear and accurate information should be provided to the family where possible, with acknowledgement of uncertainties, together with an honest appraisal of the likelihood of various potential outcomes. Decisions should be made with close involvement of the family, in the best interests of the child.

### Management of Respiratory Failure

Both the specialist team and any paediatric respiratory specialist involved in the care of infants with severe OI should have a good understanding of the multi-faceted pathophysiology of respiratory problems in OI. They should have a broad appreciation of the likely and potential long-term outcomes in severe OI. These are typically good from a neurocognitive perspective, though not always; one should recognise that the more severe the case, the more uncertain the outcome. In addition, it must be recognised that the literature is sometimes limited, with cases described as “lethal” which may or may not have been so with different care. Thus, there is risk that framing bias might lead to a self-fulfilling prophecy of lethality in some cases.

In situations where respiratory support is deemed necessary and appropriate, it is important to make sure that this is sufficient. There is a risk of undue caution allowing chronic under-ventilation, risking a vicious cycle of persistent collapse, recurrent chest infections, and gradual deterioration. In the context of severe OI, the need for respiratory support is most commonly for short-lived supplemental oxygen and/or non-invasive ventilation in the immediate days and weeks after birth. Effectiveness of non-invasive ventilation can be limited by specific difficulties such as achieving a good seal with a facemask; the weakness of bones and size of the fontanelle can mean that it is difficult to fit a facemask sufficiently well without the application of a degree of pressure that might be harmful (e.g. significant deformation of the skull vault). More prolonged supplemental oxygen and non-invasive positive-pressure ventilation may be required in some cases, and this may be necessary for months or a few years. In general, one can expect the need for ventilatory support to disappear or lessen over time as rib fractures heal, bones strengthen through the effects of bisphosphonate therapy, the chest grows, and the lungs mature. In extreme cases, adequate ventilatory support may require long-term invasive ventilation and tracheostomy. In such situations, one may anticipate this to be needed for many years.

### Medical Therapy

In a newborn with severe OI, pain management is crucial. Some form of opiate analgesia is commonly required [4•]. With multiple fractures, a continuous opiate infusion may be the best approach in the first instance, although this can be weaned once the fractures stabilise over the course of a week or so.

Intravenous bisphosphonate treatment is effective in increasing lumbar spine bone mineral density, does not impair growth, and may reduce fracture rates and help preserve vertebral heights [1, 5–8]. Whilst trial data are limited, bisphosphonates have been used in infants with OI for more than 2 decades in specialist clinical centres across the world. Over that time, because of the cumulative experience of both their efficacy and safety, bisphosphonates have become standard of care for infants with severe OI. Rather than whether to treat, most commonly, the decisions are when, with which bisphosphonate, and how.

Bisphosphonates are an effective analgesic agent [4•]. Infants can be seen to settle after a first infusion of bisphosphonate, becoming more comfortable, with basic physical observations improving. However, whilst early treatment can be helpful, any decision to treat is, of course, a balance between benefit and risk. Timing of first infusions varies. One approach is to delay treatment for a few days where possible, until the usual physiological changes in the immediate postnatal period have been completed. First infusions of bisphosphonate are well known to cause acute phase reactions. In infants with severe OI, acute cardiovascular deterioration is well recognised as a potential serious adverse effect, perhaps particularly in those infants in whom there is already some cardiorespiratory compromise [9]. An infant under the care of one of the authors, who was undergoing intensive monitoring, was seen to develop significant pulmonary hypertension during a first infusion of pamidronate which resolved with cessation of the same. We take a cautious approach of admitting all infants with severe disease to a high dependency environment for their first infusion of bisphosphonate. To mitigate the risk of a severe acute phase reaction, we also ensure an infant is not vitamin D deficient and administer simple anti-pyretic medication, typically paracetamol, for several days after the first infusion.

Hypocalcaemia is a risk following first bisphosphonate infusions. Serum calcium levels drop during infusions and for a few days afterwards. Routine administration of calcium supplements for a few days following the first infusion usually avoids any significant problems. Symptomatic hypocalcaemia is very uncommon, but families should be warned of the risk and asked to get in touch if concerned (e.g. if the infant develops an intercurrent illness following admission which may increase the chance of problems, say gastroenteritis).

It is good practice to routinely counsel about risks and concerns regarding both short-term and long-term risks of bisphosphonate treatment in OI and to provide written information about the same [10]. This should be revisited at times beyond the immediate neonatal period, as the family’s understanding grows, and perspectives change.

There is no clear consensus on dose and frequency of administration of intravenous bisphosphonates. Annual doses of pamidronate have varied from 6 mg/kg to 12 mg/kg [6, 8]. It is common to start with more frequent and lower doses initially. We routinely use a starting dose of pamidronate of 0.5 mg/kg, typically administered over 4 h (Table 1). In exceptional cases, where the risk of cardiovascular instability is deemed high or the potential consequences of it are extreme, we have administered a starting dose of 0.25 mg/kg. Other similar regimens are used elsewhere, and zoledronic acid is employed by some.

**Table 1:** Example of a guideline for doses of pamidronate to be used in treating infants with severe osteogenesis imperfecta in the first year of life (after Senthilnathan [8])

Timing–weeks after 1st cycle	Cycle	Dose (mg/kg/d)^a^
	1	0.5 mg/kg
6	2	0.75 mg/kg
13	3	1 mg/kg
21	4	1 mg/kg
30	5	1.25 mg/kg
40	6	1.5 mg/kg
52	7	1.5 mg/kg

^a^Each cycle is a single infusion on each of 2 successive days

At the present time there are no other effective bone-targeted medical or cell-based therapies available for the treatment of infants with OI.

### Vascular Access

As bisphosphonate treatment is expected to be administered long-term, it is clear that venous access will be required both for blood sampling and drug administration. Peripheral venous access can be difficult in small infants and may get more difficult over time. As well as pain and distress, there are specific risks related to holding infants with OI for insertion of a peripheral catheter such as fracture. Central venous access devices (CVADs) largely avoid these risks, although insertion itself carries risks and there is the risk of serious sepsis, albeit that this is probably low with proper care [11•]. There have been cases of infants with OI experiencing frequent infections necessitating line removal, and it has been suggested that the impact of OI on soft tissues may predispose to problems with CVADs in small and severely affected infants. In truth, there is little published evidence to guide either routine use or avoidance of CVADs in OI, and some centres insert these routinely in all severely affected infants with good results. Other centres are more cautious, inserting CVADs either when clearly indicated on the balance of risk (e.g. difficulty inserting peripheral catheters which is likely to recur) or deferring routine insertion for a few months, thereby perhaps lessening the risks.

### Orthopaedic Management

**Fig. 2:**
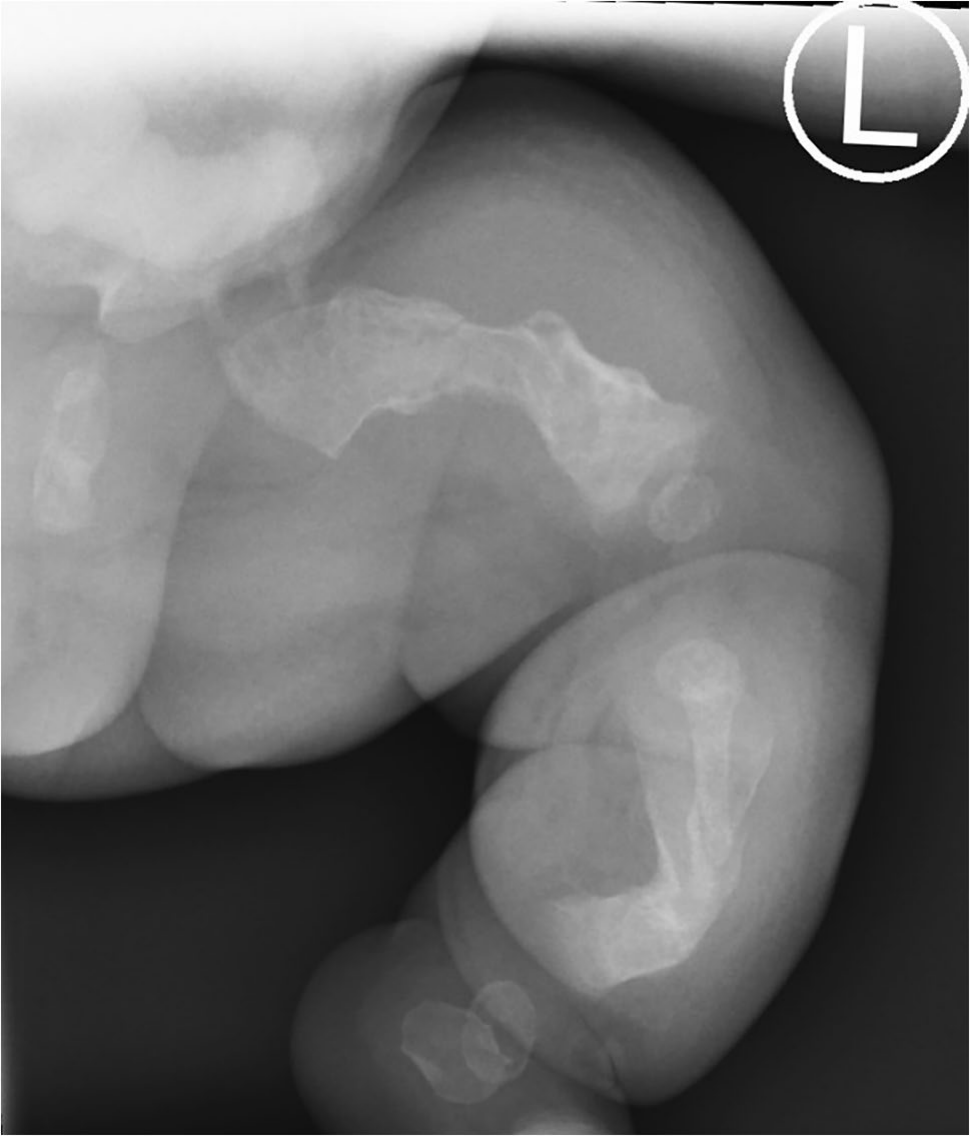
Plain radiograph of left lower limb of the same infant with severe osteogenesis imperfecta shown in Fig. 1. There are fractures as well as shortening, widening, and angulation of the femur, tibia, and fibula. These skeletal features are indicative of generalised profound material weakness and low bone mass

Infants with severe OI are commonly born with significant long bone deformities, may have multiple long bone fractures, and limbs may lie fixed in extreme positions which may hinder care and handling (Fig. 2). Orthopaedic surgery is seldom required at this early stage. However, involvement of an orthopaedic surgeon is often helpful both to advise on management of limb fractures, perhaps to set the scene for future discussions about orthopaedic care, and/or to provide reassurance that nothing further needs to be done at that stage.

Immediate management of fractures is generally conservative, with immobilisation, say with wool and crepe bandaging. Traction should not generally be used in infants with OI as it is often unnecessary and can result in harm such as further fractures.

### Feeding

Nutrition is an essential element in the care of any infant. In critically ill infants with OI who are requiring some respiratory support, it may not be possible to feed them enterally. More commonly, the dilemma is whether they can be fed orally or require a nasogastric tube. In those unfamiliar with OI there is a tendency to perceive an infant with visible physical difference and fragile bones as requiring tube feeding, particularly in the high dependency setting. It is the role of the specialist team to ensure, where appropriate, that infants are fed orally, and mothers are helped to breast feed their child [2]. Often the practical instruction of mothers is undertaken by the clinical nurse specialist, who will also educate the neonatal nursing team and serve as a point of contact during the first few days and weeks as feeding is established. In severely affected infants on oral feeds, the need to manage them in a horizontal position to minimise vertebral compression fractures (see below) can present challenges, including with “winding”, for which we commonly use of preparations containing lactase or simethicone, as well as advising on safe physical methods.

### Head Shape

The head of an infant with severe OI may feel very soft due to both the quality of the skull bones and the number of Wormian bones. Together with excessive caution in handling and, sometimes, periods of high-dependency care, plagiocephaly is common and can lead to persistent marked deformity of the skull vault. Whilst the long-term effects of marked brachycephaly and other skull and cranio-cervical junction deformities in OI may be unclear, the potential risks are sufficient to justify attention to head shape in early infancy, i.e. at a time when intervention may have an effect. The potential risks of deformity include the effect on the anatomy of the cranio-cervical junction, including basilar invagination, and the presence in some cases of structural abnormalities of the cervical spine, e.g. severe cervical kyphosis [12•, 13]. Therefore, families should be shown how to vary the position of the infant’s head, and to manage any torticollis, to minimise the degree of deformity [2]. The role of aids such as specially designed pillows or helmets is still to be established [14, 15]. Whilst basilar invagination typically develops in later childhood, and both basilar invagination and significant cervical spine anomalies are rare, there may be a role for magnetic resonance scan imaging in infancy as a baseline and/or screening assessment.

## Beyond Early Postnatal Care

### The Start of a Long-term Relationship

Very often the specialist team caring for an infant with OI will remain responsible for their care until the child transitions to services for young adults. This is desirable from the perspective of continuity of care. It can also be used as a powerful demonstration to families of the alignment of their interests with those of the clinical team, i.e. everyone is working toward the long-term interests of the child. This can be helpful in discussions regarding specific aspects of management as well as in developing trust more generally. The provision of details of patient support organisations to families is important, both to broaden families’ support and information, and to promote agency.

### Planning Discharge

The transition from hospital care to care in the home by the family is a key event, and the way it is managed is important both for the family’s relationship with their child and with healthcare services. It is an opportunity to build confidence and trust. It should be done in a planned and supportive fashion, with appropriate safeguards in place. Timing of discharge depends on the infant’s medical needs, the family’s ability to provide the care necessary to manage the child at home, and the establishment of a network of healthcare professionals who will be able to visit the child and/or who are easily contactable by the family in case of any concerns or difficulties.

In cases of respiratory and/or feeding difficulties, sufficient assessment must have taken place to ensure that an infant is not at significant risk of deterioration at home. Where supportive measures such as oxygen dependence or tube feeding are required, discharge will be delayed until specific training has taken place and discharge planning will involve multiple medical disciplines. More generally, families need to be competent and to feel confident in handling, feeding, dressing, and changing nappies. Advice should include the importance of dressing in loose clothes, how to change nappies without holding the legs, and how to position whilst feeding and sleeping. This advice should be provided by those experienced in the practical handling of infants with OI in person with hands-on guidance and the opportunity to observe the family undertaking key tasks prior to discharge [2]. This learning should be reviewed and reinforced during planned visits to the home.

Car seating and the home situation should be reviewed as part of discharge planning and support given to resolve any barriers to sending the child home. It is important that local primary and secondary care services, and sometimes social care are made aware of the discharge to home in a timely fashion and may need to be directly involved in discharge planning.

There should be a clear plan in place for regular contact with the family in the days following discharge including phone calls to see how the family are managing and feeling, and a review in the home, ideally by members of the specialist multidisciplinary team (e.g. nurse, physiotherapist, and occupational therapist) to reiterate advice already given and deliver further support and training in the home setting [2]. We routinely introduce families to a specialist psychologist during this period. We commonly involve a specialist social worker to ensure that the family can access the funding and other support which is available to them.

### Framing Management in Terms Of Long-term Goals

Important goals of clinical management are to enable a child to reach their physical, emotional, and intellectual potential, and to become as independent as they can be within society, whilst ideally enjoying the support of a network of friends and family [16••]. Getting care right in infancy is critical to achieving these goals. Infancy is a period of rapid and substantial skeletal growth as well as vulnerability in terms of risk of fracture including vertebral height loss. It is also a period of profound neurocognitive development.

Central to the goals of medical therapy are the reduction of fracture rate and minimisation of vertebral height loss/fractures. For children with a whole range of disease severities, we advise that they should not be encouraged to undertake activities for which they are not developmentally ready, say sitting without adequate head or trunk control (e.g. avoidance of early unsupported sitting and baby walkers) [2]. This is particularly the case for infants with severe disease, in whom periods of elevation may result in vertebral compression fractures. Thus, for children with severe OI, we usually advise parents to minimise periods of elevation beyond the horizontal as far as possible during early infancy. Whilst a consequence of this approach is that gross motor development may be temporarily delayed, this is with the intention of improving medium and long-term outcomes. Subsequent progress towards sitting is managed with stepwise increases in the recommended maximum degrees and duration of elevation. Decisions about such increases are made by the multidisciplinary team as a whole and take into account multiple factors, including the underlying disease, clinical examination findings, assessment of motor skills, assessment of developmental needs, and spinal imaging [2]. If a child is already able to sit then it is not appropriate to limit the maximum degree of elevation (although it is important to provide the child with suitable supportive seating and the opportunity for periods of rest in the horizontal position). Such undue restriction in elevation can result in a child repeatedly trying to sit from lying, potentially increasing the force through vertebrae at the thoraco-lumbar junction thereby increasing the risk of fractures.

Whilst temporary restrictions recommended by clinical teams may have some effect on gross motor development, for the most part it is delayed in infants with severe OI by the underlying disease, episodes of and periods of recovery from fractures, long bone deformity, pain, and episodes of illness and hospital admission. Motor development can also be limited by restrictions on activity either inadvertent or deliberate which are imposed by carers or clinicians due to understandable but excessive concern. It is important to actively identify and address unnecessary limitations that can be avoided or overcome.

Helping parents and staff facilitate neurocognitive development is an important role of the specialist clinical team. The same factors which delay and obstruct motor development may delay other aspects of development. In addition to necessary restrictions on activity, an infant may be treated differently to other children in ways that impact upon their development. An early emphasis on the importance of neurocognitive development is important in ensuring both timely provision of suitable experiences and toys to facilitate development in a relatively immobile child (e.g. switch-adapted toys to help encourage development of motor control and to learn cause and effect), and that due consideration is given to the balance that should be struck between promotion of development and other aspects of care (e.g. balancing risks of vertebral fracture with facilitation of interaction, play, and weaning in determination of timing of progression to greater degrees and longer periods of elevation with seating).

Parental mental health is an important element in ensuring a child’s optimal development. It is not uncommon for parents to experience adverse mental health effects from the experience of having had a child diagnosed with a serious illness and the profound impact that this has on their lives including their relationships. There is also the understandable apprehension and fear that can accompany caring for a child with severe bone fragility. The specialist team ought to anticipate and be vigilant for evidence of such problems. Good education and support, perhaps involving proactive involvement of social care and psychology, as well as skilled troubleshooting can be useful in helping prevent or overcome some of the difficulties encountered by families.

### Nutrition and Growth

There is a role for a dietician in the first year of life to ensure that the child is adequately nourished. Dietetic advice should be provided in conjunction with the rest of the team, particularly around weaning and introduction of finger foods.

Growth should be monitored carefully in all children with OI. A common error of non-specialist services is to aim for a rate of growth which exceeds that which one would expect for a child with severe OI. Obesity may contribute to difficulties with mobility and pain and should be avoided. Interpretation of growth data by the specialist team, and the dietician can provide reassurance to parents and local teams which may avoid unnecessary concern or dietary supplementation [17, 18]. In contrast, there may be difficulty in identifying undernourishment. It is helpful to identify infants at high risk of poor nutrition so that closer surveillance of growth can take place. For example, those with increased respiratory effort may need additional calories. Weight adjusted for height or length can be helpful in assessing children with short stature in the absence of relevant disease-specific growth charts.

As with all infants, it is important to ensure that vitamin D status is adequate to optimise skeletal mineralisation and development. Early life vitamin D deficiency is most commonly a consequence of maternal deficiency. It therefore makes sense to review the maternal vitamin D status and assess that of the infant within the first few weeks of life. Appropriate supplementation, or treatment should be initiated according to local protocols, typically aiming for a serum 25 OH vitamin D concentration of at least 50 nmol/L.

## Type 5 OI

An infant with type 5 OI presents a situation in which a precise diagnosis directs treatment beyond that which would be indicated by the clinical severity alone. Diagnosis in early infancy is possible from either family history, characteristic neonatal radiographic findings, and/or the well-described mutation in the promoter of *IFITM5* [19]. It is our experience that development of multiple vertebral fractures in the first year of life is almost universal amongst children with type 5 osteogenesis imperfecta (own observations, unpublished). In our centre, we have adopted a policy of bisphosphonate treatment of those with type 5 osteogenesis imperfecta from early infancy, alongside a cautious approach to elevation to sitting, even in the absence of fractures. We use a treatment regimen that is less aggressive than for more severe types of OI, typically 1 mg/kg 3 monthly, after a lower early starting dose. This has resulted in good outcomes with preservation of vertebral heights (own observations, unpublished).

## Conclusions

Early life management of severe OI is complex. The early involvement of multidisciplinary specialist care is critical in ensuring optimal outcomes for the infant and in setting the scene for future relationships between the family and health care services. Communication between neonatal teams and local specialist services is key to ensuring that timely and optimal care is provided. A long-term perspective which focuses on the axial, craniofacial, and peripheral skeleton as well as on development more generally provides a framework which can guide the management of infants with severe OI.
